# Supplementary material for: Resolving the full spectrum of human genome variation using Linked-Reads
Source: Genome Res. 2019 Apr;29(4):635–45. doi: 10.1101/gr.234443.118 (PMC6442396; doi:10.1101/gr.234443.118)
Supplement: Supplemental Material [file supp_gr.234443.118_Supplemental_longranger-2.2.zip › longranger-2.2.tar.bz2!/lr2.2-public/lib/stan/cmdstan/stan/doc/api/html/structstan_1_1gm_1_1expr__type.html]

Stan: stan::gm::expr\_type Struct Reference


|  |  |
| --- | --- |
|  | Stan  2.5.0  probability, sampling & optimization |


- Main Page
- Related Pages
- Namespaces
- Classes
- Files

- Class List
- Class Index
- Class Hierarchy
- Class Members

All Classes Namespaces Files Functions Variables Typedefs Enumerations Enumerator Friends Macros Pages

- stan
- gm
- expr\_type

Public Member Functions |
Public Attributes |
List of all members

stan::gm::expr\_type Struct Reference

`#include <ast.hpp>`

|  |  |
| --- | --- |
| Public Member Functions | |
|  | expr\_type () |
|  | |
|  | expr\_type (const base\_expr\_type base\_type) |
|  | |
|  | expr\_type (const base\_expr\_type base\_type, size\_t num\_dims) |
|  | |
| bool | operator== (const expr\_type &et) const |
|  | |
| bool | operator!= (const expr\_type &et) const |
|  | |
| bool | operator< (const expr\_type &et) const |
|  | |
| bool | operator<= (const expr\_type &et) const |
|  | |
| bool | operator> (const expr\_type &et) const |
|  | |
| bool | operator>= (const expr\_type &et) const |
|  | |
| bool | is\_primitive () const |
|  | |
| bool | is\_primitive\_int () const |
|  | |
| bool | is\_primitive\_double () const |
|  | |
| bool | is\_ill\_formed () const |
|  | |
| bool | is\_void () const |
|  | |
| base\_expr\_type | type () const |
|  | |
| size\_t | num\_dims () const |
|  | |

|  |  |
| --- | --- |
| Public Attributes | |
| base\_expr\_type | base\_type\_ |
|  | |
| size\_t | num\_dims\_ |
|  | |

## Detailed Description

Definition at line 74 of file ast.hpp.

## Constructor & Destructor Documentation

|  |  |  |  |  |
| --- | --- | --- | --- | --- |
| stan::gm::expr\_type::expr\_type | ( |  | ) |  |

Definition at line 53 of file ast\_def.cpp.

|  |  |  |  |  |  |
| --- | --- | --- | --- | --- | --- |
| stan::gm::expr\_type::expr\_type | ( | const base\_expr\_type | *base\_type* | ) |  |

Definition at line 57 of file ast\_def.cpp.

|  |  |  |  |
| --- | --- | --- | --- |
| stan::gm::expr\_type::expr\_type | ( | const base\_expr\_type | *base\_type*, |
|  |  | size\_t | *num\_dims* |
|  | ) |  |  |

Definition at line 61 of file ast\_def.cpp.

## Member Function Documentation

|  |  |  |  |  |
| --- | --- | --- | --- | --- |
| bool stan::gm::expr\_type::is\_ill\_formed | ( |  | ) | const |

Definition at line 105 of file ast\_def.cpp.

|  |  |  |  |  |
| --- | --- | --- | --- | --- |
| bool stan::gm::expr\_type::is\_primitive | ( |  | ) | const |

Definition at line 93 of file ast\_def.cpp.

|  |  |  |  |  |
| --- | --- | --- | --- | --- |
| bool stan::gm::expr\_type::is\_primitive\_double | ( |  | ) | const |

Definition at line 101 of file ast\_def.cpp.

|  |  |  |  |  |
| --- | --- | --- | --- | --- |
| bool stan::gm::expr\_type::is\_primitive\_int | ( |  | ) | const |

Definition at line 97 of file ast\_def.cpp.

|  |  |  |  |  |
| --- | --- | --- | --- | --- |
| bool stan::gm::expr\_type::is\_void | ( |  | ) | const |

Definition at line 108 of file ast\_def.cpp.

|  |  |  |  |  |
| --- | --- | --- | --- | --- |
| size\_t stan::gm::expr\_type::num\_dims | ( |  | ) | const |

Definition at line 114 of file ast\_def.cpp.

|  |  |  |  |  |  |
| --- | --- | --- | --- | --- | --- |
| bool stan::gm::expr\_type::operator!= | ( | const expr\_type & | *et* | ) | const |

Definition at line 70 of file ast\_def.cpp.

|  |  |  |  |  |  |
| --- | --- | --- | --- | --- | --- |
| bool stan::gm::expr\_type::operator< | ( | const expr\_type & | *et* | ) | const |

Definition at line 73 of file ast\_def.cpp.

|  |  |  |  |  |  |
| --- | --- | --- | --- | --- | --- |
| bool stan::gm::expr\_type::operator<= | ( | const expr\_type & | *et* | ) | const |

Definition at line 78 of file ast\_def.cpp.

|  |  |  |  |  |  |
| --- | --- | --- | --- | --- | --- |
| bool stan::gm::expr\_type::operator== | ( | const expr\_type & | *et* | ) | const |

Definition at line 66 of file ast\_def.cpp.

|  |  |  |  |  |  |
| --- | --- | --- | --- | --- | --- |
| bool stan::gm::expr\_type::operator> | ( | const expr\_type & | *et* | ) | const |

Definition at line 83 of file ast\_def.cpp.

|  |  |  |  |  |  |
| --- | --- | --- | --- | --- | --- |
| bool stan::gm::expr\_type::operator>= | ( | const expr\_type & | *et* | ) | const |

Definition at line 88 of file ast\_def.cpp.

|  |  |  |  |  |
| --- | --- | --- | --- | --- |
| base\_expr\_type stan::gm::expr\_type::type | ( |  | ) | const |

Definition at line 111 of file ast\_def.cpp.

## Member Data Documentation

base\_expr\_type stan::gm::expr\_type::base\_type\_

Definition at line 75 of file ast.hpp.

size\_t stan::gm::expr\_type::num\_dims\_

Definition at line 76 of file ast.hpp.

---

The documentation for this struct was generated from the following files:

- src/stan/gm/ast.hpp
- src/stan/gm/ast\_def.cpp


---

[ Stan Home Page ]

*© 2011–2014,
Stan Development Team.*
